# HERV-W ENV Induces Innate Immune Activation and Neuronal Apoptosis via linc01930/cGAS Axis in Recent-Onset Schizophrenia

**DOI:** 10.3390/ijms24033000

**Published:** 2023-02-03

**Authors:** Xuhang Li, Xiulin Wu, Wenshi Li, Qiujin Yan, Ping Zhou, Yaru Xia, Wei Yao, Fan Zhu

**Affiliations:** 1 State Key Laboratory of Virology, Department of Medical Microbiology, School of Basic Medical Sciences, Wuhan University, No. 185 Donghu Road, Wuhan 430071, China; 2 Hubei Province Key Laboratory of Allergy & Immunology, Wuhan University, Wuhan 430071, China

**Keywords:** schizophrenia, HERV-W ENV, linc01930, IFN-β, cGAS, apoptosis

## Abstract

Schizophrenia is a severe neuropsychiatric disorder affecting about 1% of individuals worldwide. Increased innate immune activation and neuronal apoptosis are common findings in schizophrenia. Interferon beta (IFN-β), an essential cytokine in promoting and regulating innate immune responses, causes neuronal apoptosis in vitro. However, the precise pathogenesis of schizophrenia is unknown. Recent studies indicate that a domesticated endogenous retroviral envelope glycoprotein of the W family (HERV-W ENV, also called ERVWE1 or syncytin 1), derived from the endogenous retrovirus group W member 1 (ERVWE1) locus on chromosome 7q21.2, has a high level in schizophrenia. Here, we found an increased serum IFN-β level in schizophrenia and showed a positive correlation with HERV-W ENV. In addition, serum long intergenic non-protein coding RNA 1930 (linc01930), decreased in schizophrenia, was negatively correlated with HERV-W ENV and IFN-β. In vitro experiments showed that linc01930, mainly in the nucleus and with noncoding functions, was repressed by HERV-W ENV through promoter activity suppression. Further studies indicated that HERV-W ENV increased IFN-β expression and neuronal apoptosis by restraining the expression of linc01930. Furthermore, HERV-W ENV enhanced cyclic GMP-AMP synthase (cGAS) and stimulator of interferon genes protein (STING) expression and interferon regulatory factor 3 (IRF3) phosphorylation in neuronal cells. Notably, cGAS interacted with HERV-W ENV and triggered IFN-β expression and neuronal apoptosis caused by HERV-W ENV. Moreover, Linc01930 participated in the increased neuronal apoptosis and expression level of cGAS and IFN-β induced by HERV-W ENV. To summarize, our results suggested that linc01930 and IFN-β might be novel potential blood-based biomarkers in schizophrenia. The totality of these results also showed that HERV-W ENV facilitated antiviral innate immune response, resulting in neuronal apoptosis through the linc01930/cGAS/STING pathway in schizophrenia. Due to its monoclonal antibody GNbAC1 application in clinical trials, we considered HERV-W ENV might be a reliable therapeutic choice for schizophrenia.

## 1. Introduction

Human endogenous retroviruses (HERVs), discovered in 1981 [1], are remnants of retroviral infection to human germline cells million years ago [2], constituting about 8% of the whole genome [3]. HERVs are regularly composed of gag, pro, pol, and env, with two long terminal repeats aside [4]. Most HERVs remain inactive due to mutation accumulation [5]. However, some HERVs still have open reading frames to encode functional transcripts and participate in various normal physiological processes, such as embryogenesis [6]. Recent studies show that HERVs derive nucleic acids or proteins involved in antiviral responses [7]. ERV-derived long noncoding RNA (lncRNA) enhances innate immune responses [8]. Furthermore, HERVs constitute a dynamic reservoir of interferon (IFN)-inducible enhancers and contribute to the evolution and amplification of the IFN transcriptional network [9].

HERVs are normally divided into three classes based on their sequence similarity in the pol region, namely class I (Gammaretrovirus), class II (Betaretrovirus), and class III (Spumaretrovirus) [10]. The HERV-W family (HERV-W) makes up nearly 1% of the human genome and belongs to the class I family. The functional viral protein HERV-W ENV, an envelope glycoprotein located at chromosome 7q21.2, is also known as ERVWE1 or Syncytin-1 [11]. HERV-W ENV mediates trophoblast fusion and plays a critical role in the maintenance of maternal immune tolerance during pregnancy [12,13]. However, a growing number of studies in recent years suggest that HERV-W ENV is relevant to numerous diseases, including autoimmune diseases [14], cancers [15,16,17], and neuropsychiatric disorders [18]. We [19,20,21,22,23] and other researchers [24,25] report an abnormal increase of HREV-W ENV in schizophrenia patients.

Schizophrenia, a severe chronic debilitating mental disorder, which usually occurs in the early twenties, affects approximately 1% of the world population, with a high risk of complications, poor clinical outcomes, and increased medical costs [26]. Increasing evidence suggests the involvement of innate immune dysregulation in the pathogenesis of schizophrenia [27,28,29].

During pathogens infection, the innate immune response acts as the first line of defense by releasing cytokines such as type I interferons (IFN-α and -β) [30]. Several articles report an increase of interferon beta (IFN-β) in the cerebrospinal fluid and prefrontal cortex of patients with schizophrenia [31,32]. Recently, a series of studies demonstrated that the cyclic GMP-AMP synthase (cGAS)–stimulator of interferon genes protein (STING) pathway, a recently discovered pathway, activates the expression of type I IFNs and plays a powerful role in innate immunity [33]. LncRNAs also play crucial roles in the innate immune response by regulating the activation and function of IFN [34]; recent research proves their involvement in the etiology of schizophrenia [35]. It is noteworthy that long intergenic non-protein coding RNA 1930 (linc01930), a novel discovered lncRNA, is identified as a schizophrenia-related lncRNA located at chromosome 1p21.3 by genome-wide association study (GWAS) [36]. However, up to now, there is no report on the role of linc01930 in physiology and diseases, especially in schizophrenia.

Both abnormal activities of HERV [19,20,21,22,23,37] and dysregulation of innate immune activation [38,39,40] have been described in the development of schizophrenia. However, there is no detailed study on the relationship between HERV abnormal activation and innate immune dysregulation in schizophrenia. Here, we found that linc01930 decreased in the serum of schizophrenia patients and had a negative correlation to HERV-W ENV. We also demonstrated an increased expression of IFN-β protein level in schizophrenia patients’ serum samples and a positive correlation with HERV-W ENV. Further analysis indicated that serum linc01930 was negatively correlated with IFN-β. A series of experiments were subsequently performed to discover the mechanism of neuronal apoptosis induced by HERV-W ENV. The results suggested that HERV-W ENV significantly increased the expression of IFN-β and caused neuronal apoptosis through the activation of the cGAS/STING signaling pathway by suppressing the expression of linc01930. Moreover, linc01930 was mainly located in the nucleus and had no ability to encode peptides. In conclusion, this study revealed a novel role of HERV-W ENV in the pathogenesis of schizophrenia. Our finding also provided novel potential serum biomarkers of schizophrenia.

## 2. Results

### 2.1. Abnormal Expression of linc01930 and IFN-β, and Correlation among HERV-W ENV, linc01930, and IFN-β in Schizophrenia

**Figure 1:**
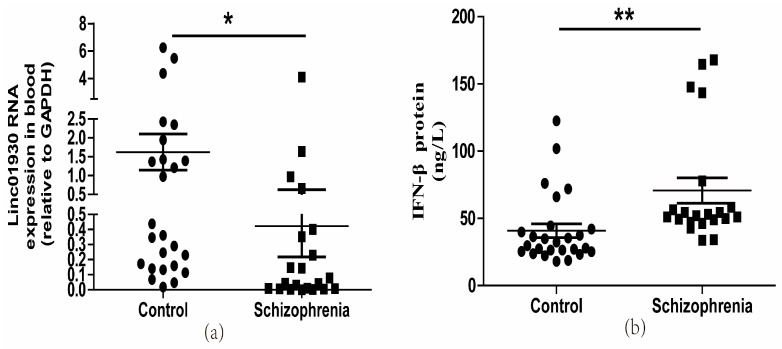

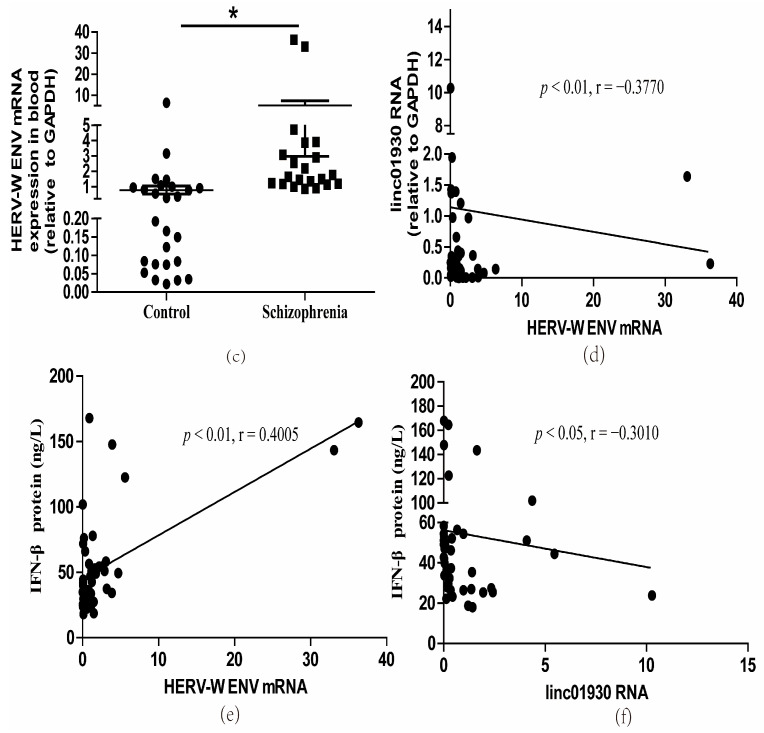
Dysregulation of HERV-W ENV, linc01930, IFN-β and their correlation analyses in schizophrenia patients. (**a**) Linc01930 RNA levels in schizophrenia patients compared with healthy controls by qRT-PCR (F, DFn, Dfd, 6.832, 25, 20). (**b**) IFN-β concentration in schizophrenia patients compared with healthy controls by ELISA (F, DFn, Dfd, 2.789, 20, 25). (**c**) HERV-W ENV mRNA levels in schizophrenia patients compared with healthy controls by qRT-PCR (F, DFn, Dfd, 54.93, 20, 25). (**d**) Correlation between HERV-W ENV and linc01930 RNA levels in schizophrenia patients and healthy controls, where Y was the RNA expression for linc01930 and X was HERV-W ENV mRNA value for each sample (F, DFn, Dfd, 0.2180, 1.000, 45.00). (**e**) Correlation between HERV-W ENV mRNA levels and IFN-β protein levels in patients with schizophrenia patients and healthy controls, where Y was the protein expression for IFN-β and X was HERV-W ENV mRNA value for each sample (F, DFn, Dfd, 26.86, 1.000, 45.00). (**f**) Correlation between linc01930 RNA levels and IFN-β protein levels in patients with schizophrenia patients and healthy controls, where Y was the protein expression for IFN-β and X was linc01930 RNA value for each sample (F, DFn, Dfd, 0.4263, 1.000, 45.00 ). Subfigures (**a**–**c**) were analyzed with Student’s *t*-test. Subfigures (**d**–**f**) were analyzed with Spearman’s rank correlation analysis. * *p* < 0.05; ** *p* < 0.01.

Serum biomarkers have been widely used to help diagnose and assess diseases’ progression [41]. It is, therefore, crucial to screen a reliable biomarker for the early detection of schizophrenia [42]. Blood-derived lncRNAs have been proposed as a new class of potential biomarkers for disease diagnosis, including cancers [43] and mental diseases [44]. A GWAS study identifies linc01930 as a susceptibility locus to schizophrenia [36]. However, there is no report on linc01930. Here, we first detected the expressions of linc01930 in the serum of 21 schizophrenia patients and 26 healthy controls. There were no significant differences in age, education level, gender, smoking status and BMI between control subjects and schizophrenia patients (Supplementary Table S1). We discovered that serum linc01930 level was decreased in schizophrenia patients compared with healthy controls (Figure 1a), with a median of 0.0466 and 0.2699, respectively (Table 1). Additionally, we found that IFN-β was increased in the blood sample of schizophrenia patients compared with healthy controls by enzyme-linked immunosorbent assay (ELISA) (Figure 1b), with a median of 52.1293 ng/L and 31.0150 ng/L, respectively (Table 2). Moreover, we also found increased HERV-W ENV at mRNA level in schizophrenia patients compared with healthy controls (Figure 1c), with a median of 1.6501 and 0.2272, respectively (Table 3). Spearman correlation analyses indicated that HERV-W ENV had a negative correlation to linc01930 (Figure 1d) and a positive correlation to IFN-β (Figure 1e), while linc01930 had a negative correlation to IFN-β (Figure 1f). In schizophrenia patients, our further analyses revealed that the consistency ratio of HERV-W ENV and linc01930 (Table 4), HERV-W ENV and IFN-β (Table 5), linc01930 and IFN-β (Table 6) was 57.1%, 66.7% and 42.8%, respectively. Thus, HERV-W ENV, linc01930, and IFN-β might be potential risk factors in schizophrenia.

### 2.2. HERV-W ENV Activated Antiviral Innate Immune Responses and Caused Neuronal Apoptosis

Our clinical data showed a positive correlation between HERV-W ENV and IFN-β in schizophrenia. The human neuroblastoma SH-SY5Y cells, which are from neuroblasts and have the potential to differentiate into neuronal cells [45], and rat primary neuronal cells, have been widely used as neuronal models of schizophrenia [20,22]. Therefore, we used SH-SY5Y and rat primary neurons to study the causal relationship between HERV-W ENV and IFN-β in neurons. Successful expression of HERV-W ENV in SH-SY5Y cells and primary neurons were shown (Supplementary Figure S1a–d).

**Figure 2:**
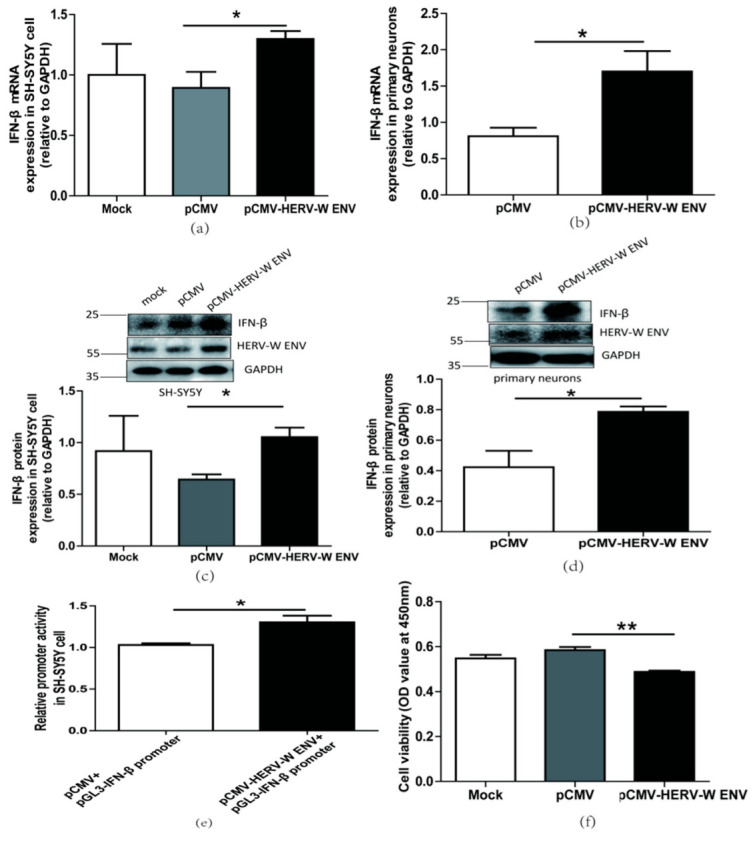

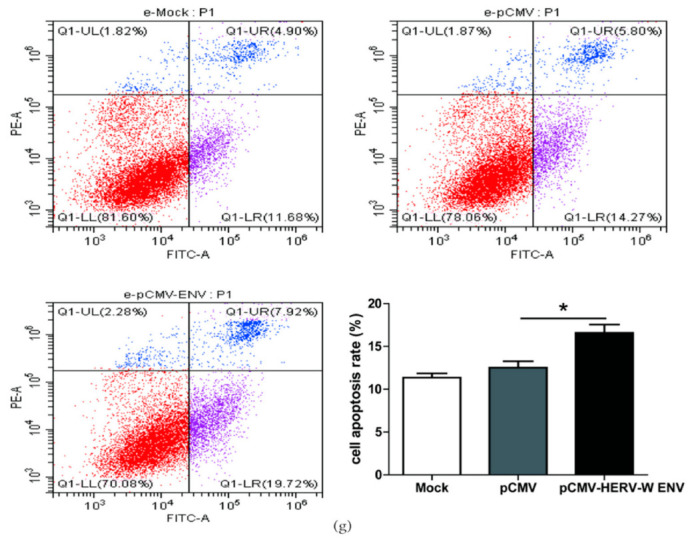
HERV-W ENV stimulated antiviral innate immune responses and mediated neuronal apoptosis (**a**,**b**) Respectively represent IFN-β mRNA levels in HERV-W ENV (0.9 μg) transfected SH-SY5Y cell (F, DFn, Dfd, 4.019, 3, 3) and rat primary neurons (F, DFn, Dfd, 5.896, 2, 2) detected by qRT-PCR. (**c**,**d**) Respectively represent IFN-β protein expression in HERV-W ENV (0.9 μg) transfected SH-SY5Y cell (F, DFn, Dfd, 3.494, 2, 2) and rat primary neurons (F, DFn, Dfd, 9.123, 2, 2) by western blotting (48 h after transfection). (**e**) Luciferase assays of pGL3- IFN-β promoter (0.2 μg) co-transfected with pCMV-HERV-W ENV (0.4 μg) in SH-SY5Y cell (F, DFn, Dfd, 3.494, 2, 2). (**f**) Cell proliferation of SH-SY5Y cell transfected with pCMV-HERV-W ENV (0.9 μg) and control vector by Cell counting kit 8 (CCK8) assays (F, DFn, Dfd, 5.892, 3, 3). (**g**) Flow cytometry analyses of HERV-W ENV (0.9 μg) on cell apoptosis in SH-SY5Y cells (F, DFn, Dfd, 1.671, 4, 4). Statistical analysis was performed by one-way analysis of variance (ANOVA). * *p* < 0.05; ** *p* < 0.01.

We found that HERV-W ENV statistically significantly increased IFN-β expression levels at the mRNA (Figure 2a,b) and protein (Figure 2c,d) in neuronal cells. Luciferase assays showed that HERV-W ENV enhanced IFN-β promoter activity in SH-SY5Y cells (Figure 2e). The production of type I interferon, including IFN-β, is the hallmark of antiviral innate immune responses [46]. So the above results indicated that HERV-W ENV activated antiviral innate immune responses in neuronal cells.

Typically, apoptotic vulnerability is increased in schizophrenia patients [47]. The type I interferon IFN-β has been reported to influence cell apoptosis [48,49,50]. The CCK8 assay results demonstrated that HERV-W ENV reduced neuronal cell proliferation (Figure 2f). Furthermore, the flow cytometry assays revealed that HERV-W ENV accelerated neuronal cell apoptosis (Figure 2g). 

In a word, HERV-W ENV evoked antiviral innate immune responses in neurons and inflated neuronal apoptosis.

### 2.3. HERV-W ENV Dowregulated the Expression of linc01930 in Neuronal Cells

**Figure 3:**
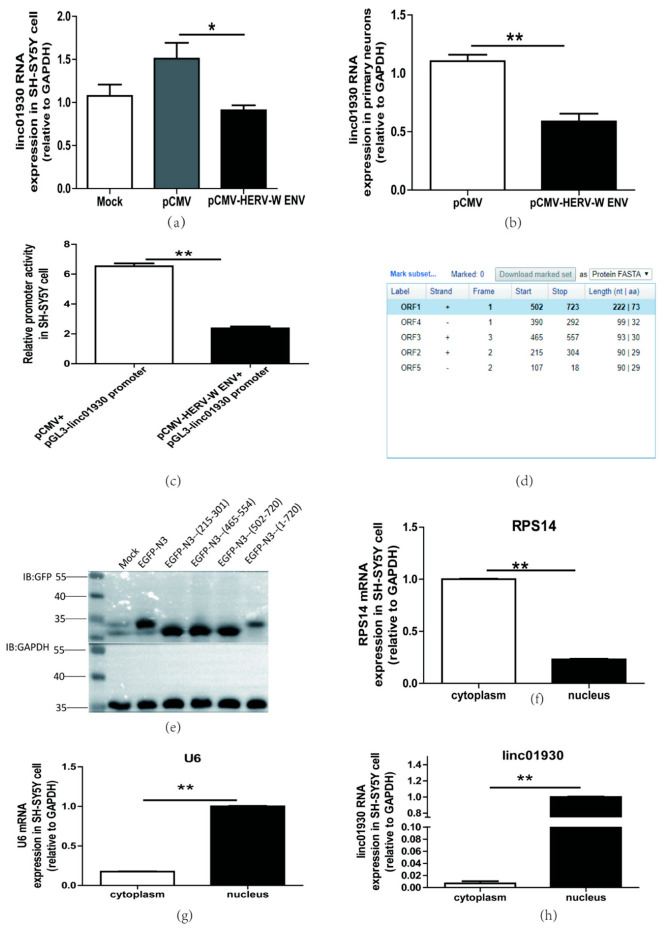
HERV-W ENV repressed linc01930 expression in neuronal cells. (**a**,**b**) Respectively represent linc01930 RNA levels in HERV-W ENV-transfected (0.6 μg) SH-SY5Y cell (F, DFn, Dfd, 10.25, 2, 2) and rat primary neurons (F, DFn, Dfd, 1.455, 2, 2) by qRT-PCR. (**c**) Luciferase assays of the pGL3-linc01930 promoter (0.2 μg) co-transfected with pCMV-HERV-W ENV plasmid (0.4 μg) in SH-SY5Y cell for 24 h (F, DFn, Dfd, 2.416, 2, 2). (**d**) Open Reading Frame (ORF) of linc01930 predicted by NCBI ORF Finder with three fragment (+215 to +304, +465 to +557, +502 to +723). (**e**) pEGFP-N3 (215-301, 465-554, 502-720, 1-720) plasmid (1.0 μg) was separately transfected in SH-SY5Y cell for 48 h and tested by western blotting. (**f**–**h**) Cellular distribution of linc01930 (F, DFn, Dfd, 2.703, 2, 2) was mainly located at the nucleus in the SH-SY5Y cell. Nuclear and cytoplasmic separation effects were quantified to RPS14 (F, DFn, Dfd, 1.316, 2, 2) in the cytoplasmic part and U6 (F, DFn, Dfd, 2.941, 2, 2) in the nuclear part. Statistical analysis was performed by one-way analysis of variance (ANOVA). * *p* < 0.05; ** *p* < 0.01.

Our clinical data suggested that HERV-W ENV was negatively correlated to linc01930 in schizophrenia patients. LncRNAs act as key regulators in brain disorders, including schizophrenia [51]. Our results from in vitro and in vivo studies showed that HERV-W ENV prominently impaired linc01930 expression in neuronal cells (Figure 3a,b). Promoters serve as a kind of “On” switch to initiate the biological process of transcription for the genes [52]. Luciferase assays indicated that HERV-W ENV markedly reduced linc01930 promoter activity in SH-SY5Y cells (Figure 3c), suggesting that HERV-ENV repressed linc01930 expression through its promoter.

As for the lack of a functional Open Reading Frame (ORF), LncRNAs can not encode proteins. However, several recent reports indicate that some lncRNAs take part in the pathogenesis of disease with their encoded peptides [53]. We found three open-reading frame fragments in NCBI ORF Finder (Figure 3d) and constructed the fragment separately in the pEGFP-N3 plasmid. The western blot analyses indicated that linc01930 did not encode peptides (Figure 3e). 

LncRNAs have diverse functions depending on their cellular localization [54]. Our results indicated that linc01930 was mainly located in the nucleus, implying that linc01930 could regulate underlying target expression at the transcriptional level (Figure 3f–h). 

Together, linc01930, suppressed by HERV-W ENV through the promoter activity, was mainly located at the nucleus and did not code peptide.

### 2.4. Linc01930 Suppressed the Antiviral Innate Immune and Neural Apoptosis Caused by HERV-W ENV

**Figure 4:**
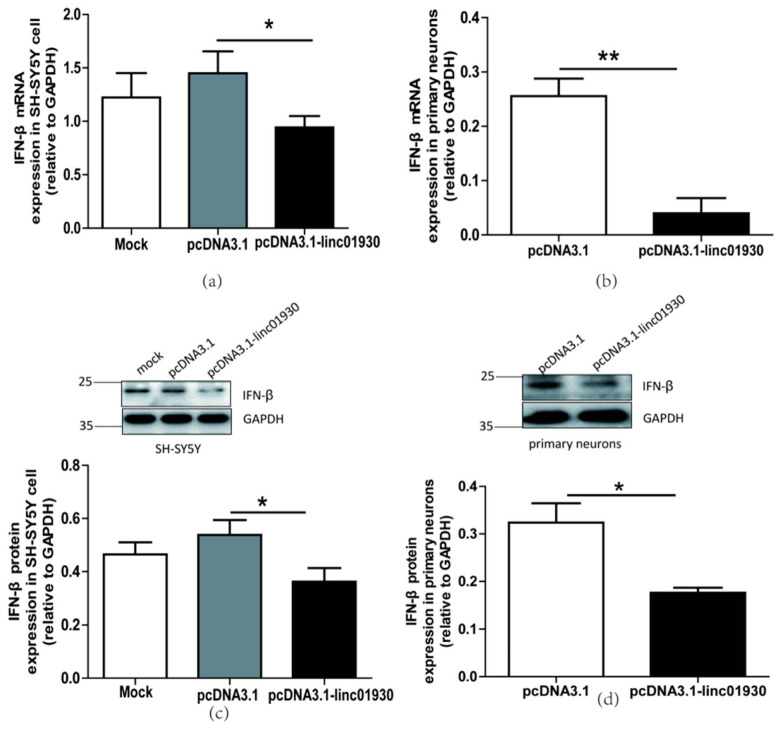

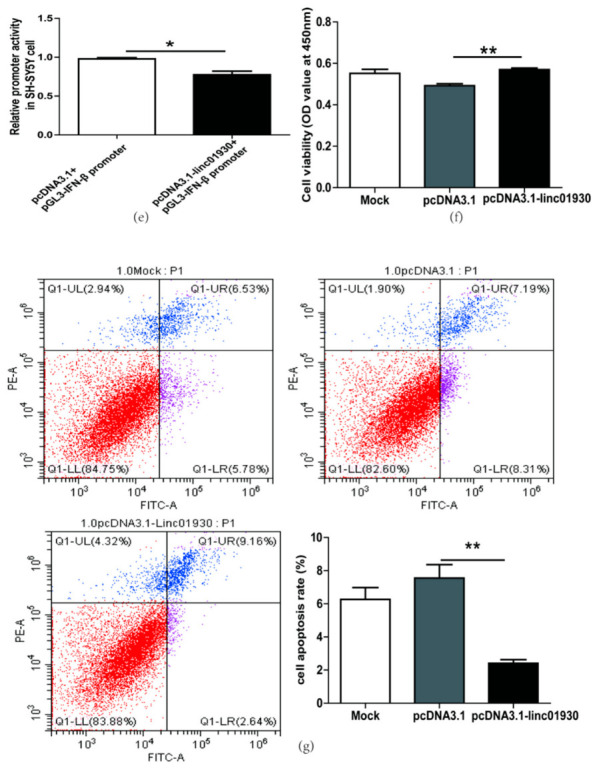
Linc01930 diminished antiviral innate immune reaction and attenuated neural cell apoptosis. (**a**,**b**) Respectively represent IFN-β mRNA levels in linc01930 (0.6 μg)-transfected SH-SY5Y cell (F, DFn, Dfd, 3.647, 11, 11) and rat primary neurons (F, DFn, Dfd, 1.305, 2, 2) by qRT-PCR. (**c**,**d**) Respectively represent IFN-β protein expression in linc01930 (0.6 μg)-transfected SH-SY5Y cell (F, DFn, Dfd, 1.196, 4, 4) and rat primary neurons (F, DFn, Dfd, 1.054, 2, 2) by western blotting. (**e**) Luciferase assays of the pGL3-IFN-β promoter (0.2 μg) co-transfected with a pcDNA3.1-linc01930 plasmid (0.2 μg) or control vector in SH-SY5Y cell (F, DFn, Dfd, 11.37, 2, 2). (**f**) Cell proliferation of SH-SY5Y cell transfected with a pcDNA3.1-linc01930 plasmid (0.6 μg) or control vector by CCK8 assays (F, DFn, Dfd, 1.555, 4, 4). (**g**) Flow cytometry analyses of 0.6 μg linc01930 on cell apoptosis in SH-SY5Y cells (F, DFn, Dfd, 13.68, 3, 3). Statistical analysis was performed by one-way analysis of variance (ANOVA). * *p* < 0.05; ** *p* < 0.01.

Several studies suggest that lncRNAs regulate innate immune response [55]. Our clinical data indicated a negative correlation between linc01930 and IFN-β in schizophrenia. However, there is no report about the effect of linc01930 on IFN-β. Efficient expression of linc01930 in neuronal cells was confirmed at the mRNA level (Supplementary Figure S2a,b). We found that linc01930 led to noticeable reductions in the mRNA (Figure 4a,b) and protein levels (Figure 4c,d) of IFN-β in neuronal cells. Furthermore, luciferase assays suggested that linc01930 inhibited IFN-β promoter activity (Figure 4e). Combined with the role of IFN-β, these results implied that linc01930 impaired antiviral innate immune response.

Some lncRNAs regulate cell apoptosis and influence disease pathogenesis [56]. The biological function of linc01930 has been ambiguous till up to now. In this article, we first reported that linc01930 increased cell proliferation (Figure 4f) and decreased apoptosis (Figure 4g) in SH-SY5Y cells. These findings denoted that linc01930 attenuated neuronal apoptosis by suppressing IFN-β.

**Figure 5:**
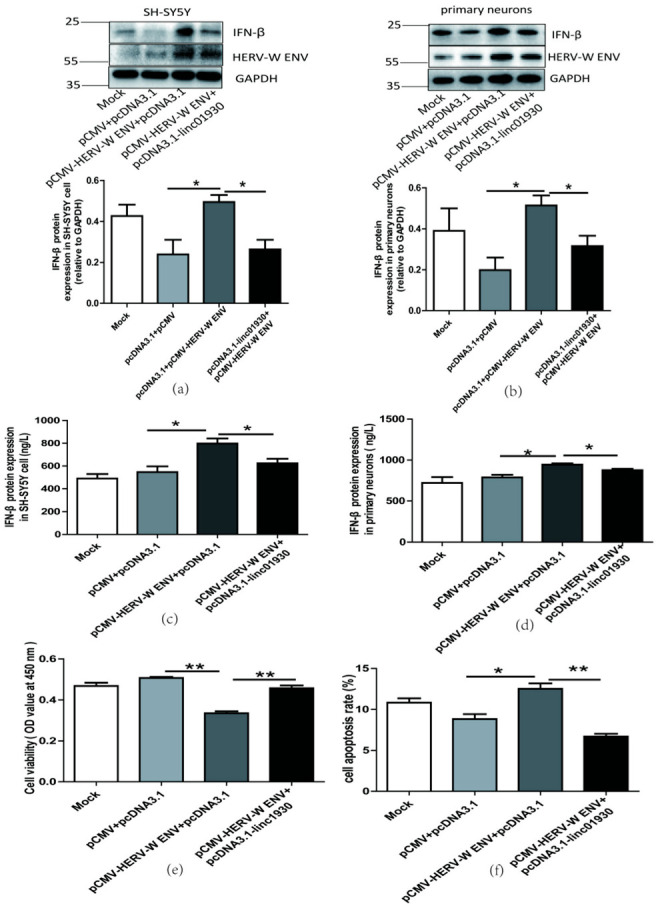

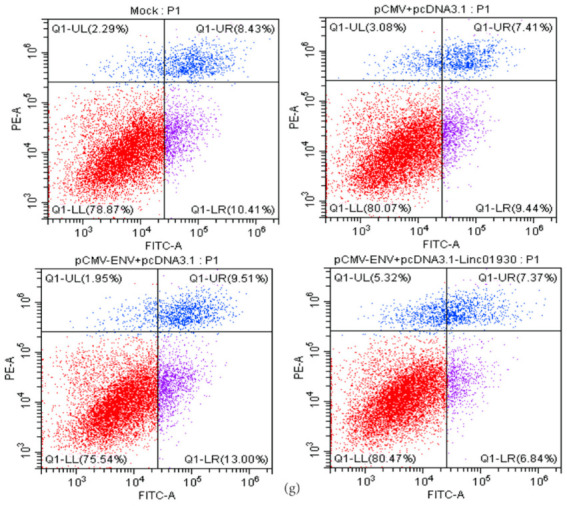
Linc01930 reversed antiviral innate immune dysfunction and neural apoptosis mediated by HERV-W ENV. (**a**,**b**) Respectively represent IFN-β protein expression after co-transfection with HERV-W ENV (0.8 μg) and linc01930 (0.4 μg) in SH-SY5Y cell (F, DFn, Dfd, 4.515, 2, 2) and rat primary neurons (F, DFn, Dfd, 1.538, 2, 2) with western blotting. (**c**,**d**) Respectively represent IFN-β expression levels after co-transfection with HERV-W ENV (0.8 μg) and linc01930 (0.4 μg) in SH-SY5Y cell (F, DFn, Dfd, 1.324, 2, 2) and rat primary neurons (F, DFn, Dfd, 4.356, 2, 2) with ELISA. (**e**) Cell proliferation was examined in SH-SY5Y cells with co-transfection of HERV-W ENV (0.8 μg) and linc01930 (0.4 μg) using the CCK8 assays (F, DFn, Dfd, 3.190, 2, 2). (f) The effect of co-transfection of HERV-W ENV and linc01930 on cell apoptosis ratios in SH-SY5Y cell were analyzed (F, DFn, Dfd, 3.641, 2, 2). (**g**) Cell apoptosis was detected in SH-SY5Y cell with co-transfection of HERV-W ENV (0.8 μg) and linc01930 (0.4 μg) using the flow cytometry. Statistical analysis was performed by one-way analysis of variance (ANOVA). * *p* < 0.05; ** *p* < 0.01.

Western blotting (Figure 5a,b) and ELISA (Figure 5c,d) indicated that linc01930 could deteriorate the increased IFN-β production stimulated by HERV-W ENV in neuronal cells. The efficient transfection of HERV-W ENV and linc01930 was shown (Supplementary Figure S5a–d). Furthermore, we found that linc01930 reversed the decreased cell proliferation caused by HERV-W ENV (Figure 5e) and markedly lessened cell apoptosis rate increased by HERV-W ENV (Figure 5f,g) in SH-SY5Y cells. Together, these results suggested that linc01930 impaired antiviral innate immune responses and neuronal apoptosis mediated by HERV-W ENV.

### 2.5. Linc01930 is Involved in the cGAS-Mediated Antiviral Signaling Pathway Activated by HERV-W ENV

**Figure 6:**
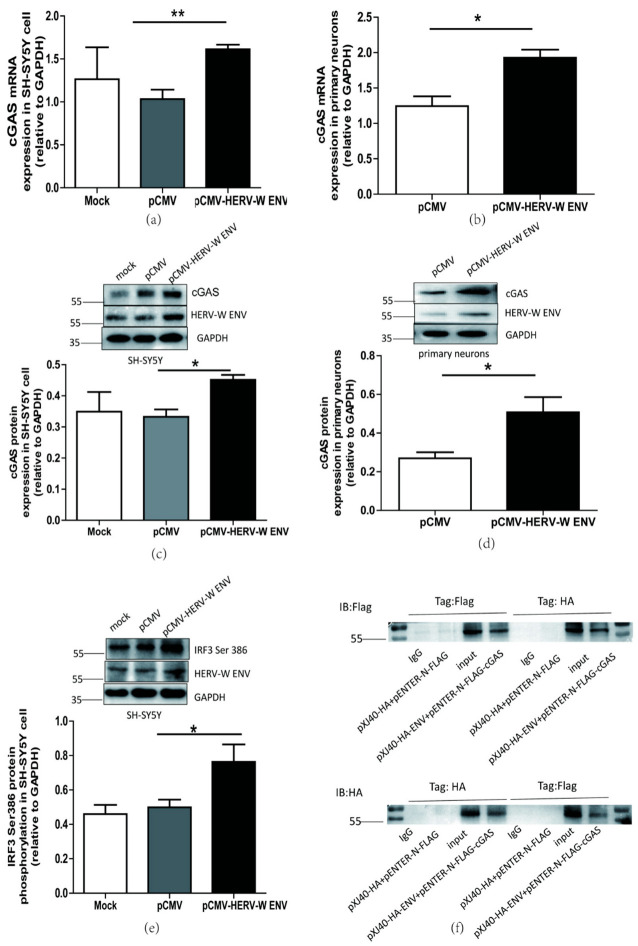
HERV-W ENV activated cGAS-mediated antiviral signaling pathway. (**a**,**b**) Respectively represent cGAS mRNA levels in pCMV-HERV-W ENV (0.9 μg) transfected SH-SY5Y cell (F, DFn, Dfd, 4.199, 2, 2) and primary neurons (F, DFn, Dfd, 1.502, 2, 2) using qRT-PCR. (**c**,**d**) Respectively represent cGAS protein expression in pCMV-HERV-W ENV (0.9 μg) transfected SH-SY5Y cell (F, DFn, Dfd, 2.168, 2, 2) and primary neurons (F, DFn, Dfd, 6.156, 2, 2) using western blotting. (**e**) HERV-W ENV (0.9 μg) effect on IRF3 phosphorylation at Ser 386 site in the SH-SY5Y cell with western blotting (F, DFn, Dfd, 5.029, 4, 4). (**f**) Co-immunoprecipitation assays (Co-IP) were performed between pXJ40-HA-HERV-W ENV (5.0 μg) and pENTER-N-FLAG-cGAS (5.0 μg) with anti-Flag and anti-HA magnetic beads by western blotting in HEK-293T cell. Statistical analysis was performed by one-way analysis of variance (ANOVA). * *p* < 0.05; ** *p* < 0.01.

Exogenous retroviruses trigger cGAS-dependent IFN-β production and innate immune response [57]. There is no report about the impact of endogenous retroviruses (ERVs) on cGAS. Here we found that HERV-W ENV substantially elevated mRNA expression of cGAS (Figure 6a,b) and STING (Supplementary Figure S3a,b) in neuronal cells. Consistently, western blot analyses showed HERV-W ENV mediated higher levels of cGAS (Figure 6c,d) and STING (Supplementary Figure S3c,d) in neuronal cells. Interferon regulatory factor 3 (IRF3) phosphorylation at Ser 386 sit is essential to cGAS-induced IFN-β expression [58]. The western blotting indicated that HERV-W ENV enhanced the phosphorylation of IRF3 (Figure 6e), suggesting that HERV-W ENV triggered the cGAS signaling pathway. Co-IP analyses indicated that HERV-W ENV interacted with cGAS (Figure 6f). Together, we found that HERV-W ENV interacted with cGAS and stimulated cGAS–STING axis through IRF3 phosphorylation. 

**Figure 7:**
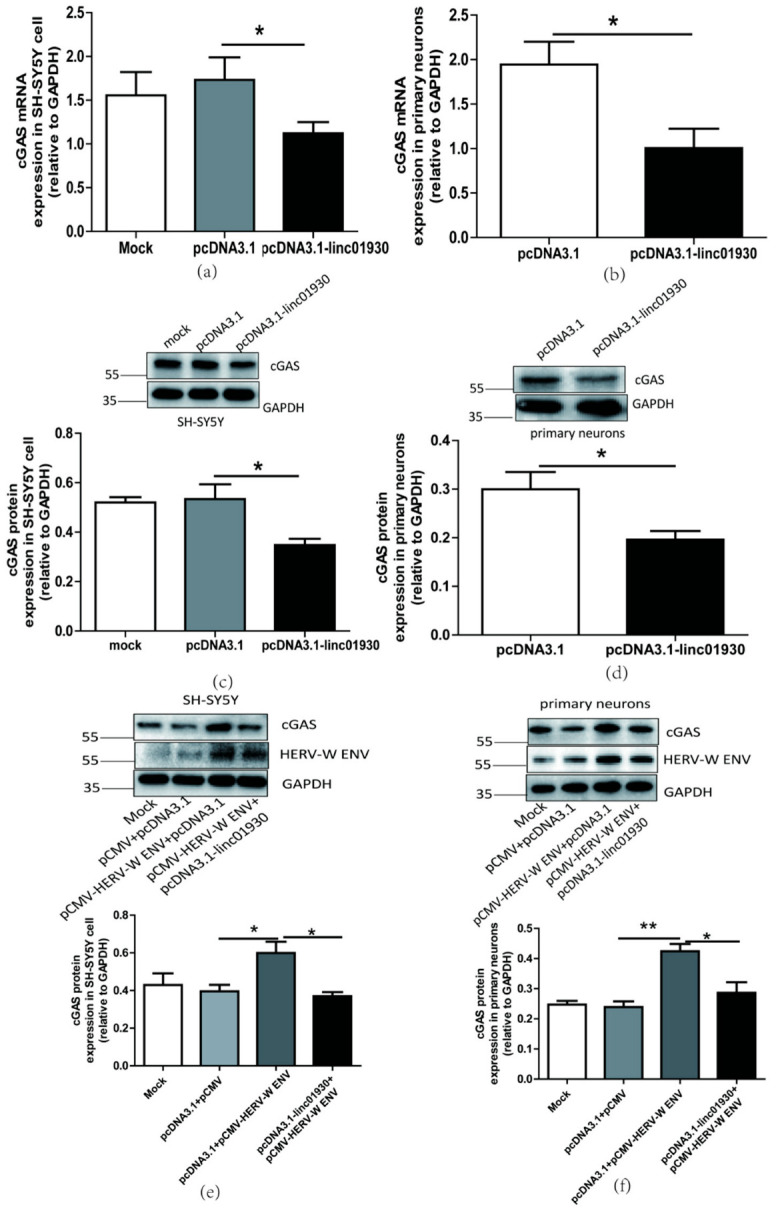
Linc01930 impaired the cGAS-mediated antiviral signaling pathway mediated by HERV-W ENV in neuronal cells. (**a**,**b**) Respectively represent cGAS mRNA levels in pcDNA3.1-linc01930 (0.6 μg) transfected SH-SY5Y cell (F, DFn, Dfd, 4.174, 8, 8) and primary neurons (F, DFn, Dfd, 1.365, 2, 2) using qRT-PCR. (**c**,**d**) Respectively represent cGAS protein expression in pcDNA3.1-linc01930 (0.6 μg) transfected SH-SY5Y cell (F, DFn, Dfd, 5.492, 2, 2) and primary neurons (F, DFn, Dfd, 4.058, 3, 3) using western blotting. (**e**) cGAS protein levels after co-transfection of pCMV-HERV-W ENV (0.8 μg) and pcDNA3.1-linc01930 (0.4 μg) in SH-SY5Y cell with western blotting (F, DFn, Dfd, 7.053, 2, 2). (**f**) cGAS protein levels after co-transfection of pCMV-HERV-W ENV (0.8 μg) and pcDNA3.1-linc01930 (0.4 μg) in primary neurons using western blotting (F, DFn, Dfd, 2.227, 2, 2). Statistical analysis was performed by one-way analysis of variance (ANOVA). * *p* < 0.05; ** *p* < 0.01.

Linc01930 repressed the production of cGAS (Figure 7a–d) and STING mRNA expression (Supplementary Figure S4a–d) in neuronal cells. Further research investigated that linc01930 suppressed the cGAS signaling pathway induced by HERV-W ENV in neuronal cells (Figure 7e,f), revealing that linc01930 participated in cGAS signaling pathway activation mediated by HERV-W ENV.

### 2.6. cGAS-Mediated Antiviral Signaling Pathway is Necessary for the Antiviral Innate Immune Responses and Neuronal Apoptosis Caused by HERV-W ENV

**Figure 8:**
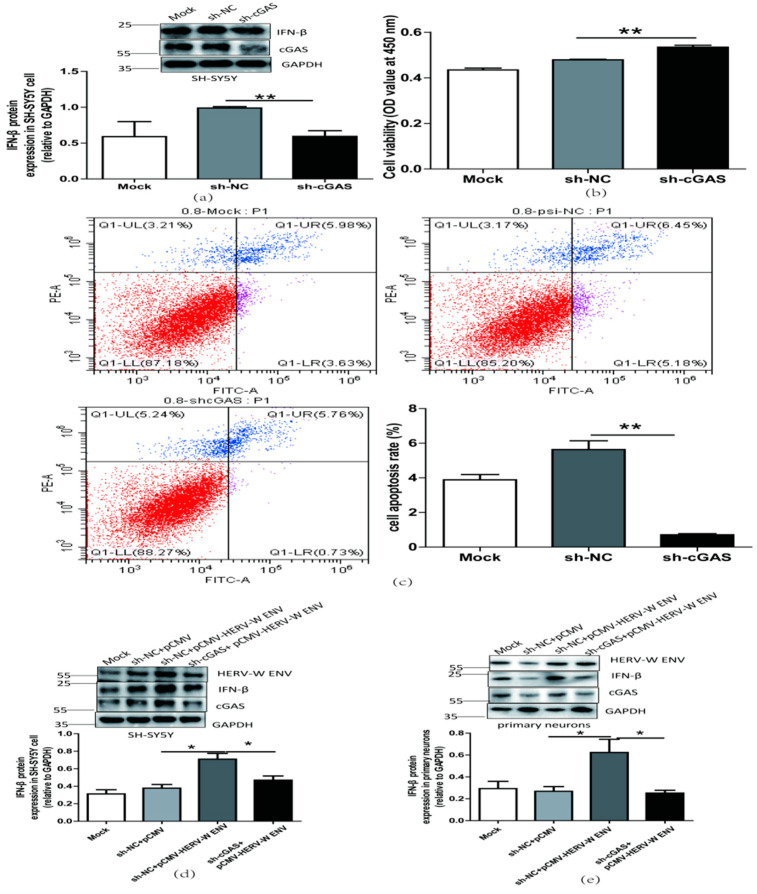

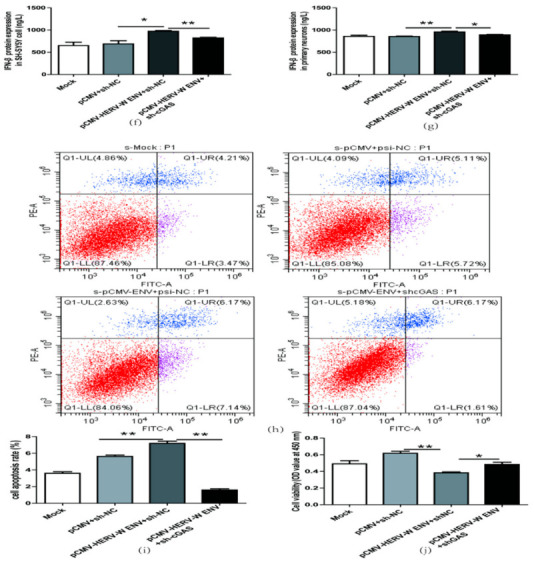
HERV-W ENV induced antiviral innate immune responses, and neural apoptosis depended on the cGAS-mediated antiviral signaling pathway. (**a**) IFN-β levels in a transfected sh-cGAS plasmid (0.9 μg) in SH-SY5Y cell using western blotting (F, DFn, Dfd, 16.70, 2, 2). (**b**) Cell proliferation of SH-SY5Y cell transfected with a sh-cGAS plasmid (0.9 μg) performed with CCK8 test (F, DFn, Dfd, 11.18, 4, 4). (**c**) Cell apoptosis of SH-SY5Y cell transfected with sh-cGAS plasmid performed with the flow cytometry (F, DFn, Dfd, 58.90, 3, 3). (**d**,**e**) Respectively represent IFN-β protein expression after co-transfection with HERV-W ENV (0.8 μg) and sh-cGAS (0.6 μg) in SH-SY5Y cell (F, DFn, Dfd, 7.053, 2, 2) and rat primary neurons (F, DFn, Dfd, 7.053, 2, 2) with western blotting. (**f**,**g**) Respectively represent IFN-β expression levels after co-transfection with HERV-W ENV(0.8 μg) and sh-cGAS (0.6 μg) in SH-SY5Y cell (F, DFn, Dfd, 13.28, 2, 2) and rat primary neurons (F, DFn, Dfd, 2.392, 2, 2) with ELISA. (**h**) Cell apoptosis was detected in SH-SY5Y cell with co-transfection of HERV-W ENV (0.8 μg) and sh-cGAS (0.6 μg) using the flow cytometry. (i) The effect of co-transfection of HERV-W ENV and sh-cGAS on cell apoptosis ratios in SH-SY5Y cell were analyzed (F, DFn, Dfd, 2.650, 2, 2). (**j**) Cell proliferation was examined in SH-SY5Y cells with co-transfection of HERV-W ENV (0.8 μg) and sh-cGAS (0.6 μg) using CCK8 (F, DFn, Dfd, 4.019, 2, 2). Statistical analysis was performed by one-way analysis of variance (ANOVA). * *p* < 0.05; ** *p* < 0.01.

cGAS promotes IFN-β production and mediates innate immune response [46]. Our results also found that the knockdown of cGAS decreased IFN-β expression at the protein level (Figure 8a), increased cell proliferation (Figure 8b) and decreased cell apoptosis (Figure 8c) in SH-SY5Y cells. Successful knockdown of cGAS was shown (Supplementary Figure S6a). Further studies showed that cGAS knockdown lessened the increased level of IFN-β (Figure 8d–g), elevated neuronal apoptosis (Figure 8h,i), and reduced cell proliferation(Figure 8j) mediated by HERV-W ENV in neuronal cells. Efficient transfection and successful knockdown of cGAS were shown (Supplementary Figure S6b–e). Taken together, these results demonstrated that HERV-W ENV stimulated the antiviral immune response and accelerated neuronal apoptosis through the cGAS signaling pathway.

## 3. Discussion

Schizophrenia occurs due to an interaction between environmental and genetic factors [59]. Genetic factors are the major cause [60]. An increasing number of clues indicate that HERV-W ENV, as an endogenous retroviral envelope protein and key regulator in the development of the human placenta, typically functions as a key link between genetic and environmental factors [19,20,21,22,23]. Environmental factors, including chemicals (aspirin [61], caffeine [61], cupric ion [62], hydroquinone (HQ) [63], and silver nanoparticles [64]), parasites [65] and viruses infections (Epstein–Barr virus (EBV) [66], cytomegalovirus (CMV) [67], coxsackievirus-B4 (CV-B4) [68], dengue virus serotype 2 (DENV-2) [69], human herpesvirus 6A (HHV-6A) [70], influenza A [71], Hepatitis B Virus(HBV) [72], human immunodeficiency virus 1 (HIV-1) [73], herpes simplex virus 1 (HSV-1) [74],or Severe Acute Respiratory Syndrome Coronavirus 2 (SARS-CoV-2)) [75], and smoking [76], can activate the transcription of HERV-W ENV. Abnormal expression of HERV-W ENV may directly or indirectly implicate the pathogenesis of several diseases [14,15,19], including schizophrenia [19,20,21,22,23]. Our previous studies suggest that schizophrenia patients exhibit an abnormal expression of HERV-W ENV. Our in-depth investigations reveal that HERV-W ENV contributes to the development of schizophrenia through multiple mechanisms: activating neuroinflammation via enhancing TLR3 signal [20], increasing proinflammatory cytokines’ release [77], inducing the production of nitric oxide(NO) [78], and promoting Cytotoxic T lymphocyte (CTL) responses [79] in astrocytes and microglia; regulating the expression of schizophrenia-associated genes via elevation of Ser9 phosphorylation of glycogen synthase kinase 3β (GSK3β) [80] or increasing phosphorylated cAMP responsive element binding protein (CREB) levels [19], such as brain-derived neurotrophic factor (BDNF) [19] and disrupted-inschizophrenia1 (DISC1) [81]; opening Na^+^ [23], K^+^ [23,82], and Ca^2+^ [81] ion channels; triggering abnormal dopaminergic neuron process via DRD2 [22] and DRD3 [19]; impairing neuronal energy metabolism by inhibiting complex I activity [21]. In this study, our clinical data found that linc01930, a potential serum-based biomarker, displayed a negative correlation with HERV-W ENV. Our in-depth study suggested that HERV-W ENV induced innate immune activation and mediated neuronal apoptosis through Linc01930/cGAS/IFN-β axis in the pathophysiology of schizophrenia.

The current diagnosis of schizophrenia relies on the experience of the doctor and can lead to misdiagnosed results [83]. Therefore, the efficient and early detection of biomarkers is necessary to offer a reliable way for a schizophrenia diagnosis. To our knowledge, there is no blood marker available for schizophrenia because of the blood-brain barrier [84]. Considering the fact that lncRNAs participate in neuropsychiatric disorders and easily pass through the blood –brain barrier [85], they may be suitable blood markers for neuropsychiatric disorders, including schizophrenia [83]. Some lncRNAs, such as Gomafu and AK096174, have been supposed to be potential blood biomarkers in cancers [86,87]. Nevertheless, no clinical trials of lncRNAs have been documented in schizophrenia. Bioinformatic data indicates that linc01930 is a novel susceptible locus for schizophrenia [36]. There are only a few reports that disclose the abnormal expression of linc01930 in pheochromocytoma and paraganglioma [88], and neuroblastoma [89]. The role of linc01930 in the etiology of schizophrenia remains unclear.

In this paper, we first reported that linc01930 was decreased in schizophrenia, suggesting that serum linc01930 might be a novel potential blood marker and risk factor for schizophrenia. The type I interferon IFN-β is the essential mediator of innate immunity [90]. Our clinical data showed that IFN-β was increased in the blood samples of schizophrenia. This is consistent with the reports of Volk et al. [31] and Hidese et al. [32] on brain tissue. These findings displayed IFN-β as a potential blood biomarker. Together, linc01930 and IFN-β might be new potential biomarkers for a schizophrenia diagnosis. The cut point between schizophrenia patients and healthy controls might not be significantly obvious, largely attributed to the small sample size. In addition, healthy controls possibly had a low level of linc01930 and a high level of IFN-β to show false positive results, for example, the clinical use of alpha-fetoprotein in live cancer [91]. Although the correlations among HERV-W ENV, linc01930 and IFN-β were moderately relevant, the consistency ratio of HERV-W ENV to linc01930 and IFN-β was 57.1% and 66.7%, respectively, indicating more samples possibly improved cut point of the linc01930 and IFN-β between schizophrenia patients and healthy controls, which was our aim in the further study.

Further analyses suggested that linc01930 was negatively correlated with HERV-W ENV in the serum of schizophrenia. In vitro experiments indicated that HERV-W ENV suppressed linc01930 expression in neuronal cells via promoter activity. Subcellular localization of lncRNAs has valuable clues for their molecular functions [54]. Our data demonstrated that linc01930 was mainly located in the nucleus and unable to encode function peptide, indicating it might play a role as a transcriptional regulator. As far as we know, the biological function of linc01930 remains unclear. We found that linc01930 exerted opposite effects on IFN-β expression through repressing promoter activity. Further studies manifested that linc01930 restrains neuronal apoptosis and exerts a cell proliferation role via inactivating IFN-β. From these, we could conclude that linc01930 might restrain innate immune activation and facilitate neural cell proliferation.

Our clinical data also suggested that IFN-β, increased in the blood sample of schizophrenia patients, had a positive correlation with HERV-W ENV. IFN-β, the type I interferon, is a vital mediator in innate immune activation, which functions to modulate cell growth and influence the activation of various immune cells [9]. Quite a few reports describe innate immune imbalances in schizophrenia [28,31]. In addition, several studies, including GWAS [92], support the role of innate immune activation in schizophrenia [93]. Notably, HERVs and their transcripts actively participate in innate immunity [94] and regulate the antiviral interferon network integrating into or near immune-related genes [95]. In addition, HERV insertions may lead to the amplification of IFN transcription [9]. Our cellular experiments revealed that HERV-W ENV stimulated IFN-β expression via promoter activity, suggesting that HERV-W ENV may induce antiviral innate immune responses in schizophrenia.

A recent article reports that IFN-β exerts apoptotic activity by increasing p38 MAPK activity, MK2 impulse, and HSP27 phosphorylation in SH-SY5Y cells [48]. In addition, IFN-β aggravates neuronal damage by inhibiting neuronal survival and neurite outgrowth through BDNF/TrkB axis [50]. Furthermore, IFN-β provokes the neurotoxicity directly via JAK/STAT and PI3K/AKT pathway in SH-SY5Y cell and rat primary neurons, causing cytochrome C release and intrinsic apoptotic pathway activation [49]. There is an increased susceptibility to apoptosis in Schizophrenia. The anti-apoptotic membrane-bound protein Bcl2 is decreased in the cortical of schizophrenia [96], and Bax/Bcl2 ratio is significantly higher in schizophrenia patients [97]. All these reports indicate that cell apoptosis is dysregulated in schizophrenia, which possibly leads to neuronal damage [47]. In this paper, we found that HERV-W ENV stimulated neuronal apoptosis through IFN-β. In a word, HERV-W ENV mediated neuronal apoptosis, which possibly functions in the pathogenesis of schizophrenia. An additional study demonstrated that Linc01930 repressed innate antiviral immunity and neuronal apoptosis mediated by HERV-W ENV. Together, HERV-W ENV led to neuronal damage through IFN-β via inhibiting linc01930.

Several signaling pathways, including cGAS/STING pathway, regulate the expression of IFN-β and induce innate antiviral immunity [46]. cGAS/STING induces IFN-β expression through IRF3 phosphorylation [98]. As a cytosolic DNA sensor, cGAS also mediates immune activation by HIV and other retroviruses [57]. A present study unveils that HERV-K (HML-2) stimulates interferon via cGAS/STING in COVID-19 patients [99]. Our previous work notices that HERV-W ENV triggers immune response activation through TLRs [20,77]. However, there is no report about the effect of HERV-W ENV on cGAS. In this paper, we found that HERV-W interacted with cGAS and triggered the activation of cGAS and STING in neuronal cells. Linc01930 suppressed the increased cGAS mediated by HERV-W ENV. Our in-depth study reveals that cGAS is involved in innate antiviral immunity and neuronal apoptosis induced by HERV-W ENV.

GNbAC1, a humanized IgG4 monoclonal antibody specifically interacting with HERV-W ENV [100], has been used in a one-year phase 2b clinical trial for multiple sclerosis [101]. Additionally, GNbAC1 also has favorable prospects in clinical trials for immune-related patients, such as type 1 diabetes (T1D) [102]. Our results promulgated that HERV-W ENV might be a potential target for clinical treatment in schizophrenia. Thus, a monoclonal antibody to HERV-W ENV may be significant as a novel therapy for schizophrenia treatment.

## 4. Materials and Methods

### 4.1. Clinical Blood Samples

All 21 schizophrenia patients and 26 healthy controls were recruited from Renmin Hospital, Wuhan University (Wuhan, China). The recent onset patients were diagnosed due to the Diagnostic and Statistical Manual of Mental Disorders (DSM-IV) without psychotropic drug treatment before. The healthy volunteers all passed the physical examination. The blood samples were divided into two-part, one for the RT-PCR test and the other for the ELISA test with the supernatants by centrifugation at 4 °C. Samples were stored at −80 °C before use. All subjects were informed of the notification from the Institutional Review Board of Wuhan University, School of Medicine. There were no significant differences in median age, education, BMI (body mass index), smoking habit, and sex between healthy individuals and patients. Details are listed in Supplementary Table S1.

### 4.2. Plasmid Construction

The human pCMV-HERV-W ENV plasmid was constructed due to the method mentioned before [20]. The human linc01930 (NR_146275) plasmid was cloned into the pcDNA3.1 plasmid. Three segments (+215 to +301, +465 to +554, +502 to +720, +1 to +720) of linc01930 were amplified and cloned into the pEGFP-N3 plasmid to test their encoding potential. In addition, linc01930 (−1400 to +100) promoter sequences were amplified and inserted into the pGL3-Basic plasmid separately. The human IFN-β promoter sequence (−187 to +100) was also inserted into the pGL3-Basic plasmid. Furthermore, The short hairpin RNAs targeting the cGAS (shcGAS, 5′-GGAAGGAAATGGTTTCCAA-3′) and the control shRNA (sh-NC, 5′-CAATCCTCGATCATCTGAGTC-3′) was cloned into pSilencer 2.1-U6 neo plasmid. Moreover, the CDS region of human cGAS (NM_138441) and HERV-W ENV (NM_001130925) were amplified and inserted into the pENTER-N-FLAG and pXJ40-HA plasmid, respectively. All primers were designed by oligo7 and listed in Supplementary Table S2.

### 4.3. Cell Culture and Transfection

The neuroblastoma cell line SH-SY5Y was purchased from American Type Culture Collection. The cells were maintained in the culture media of Minimal Essential Medium Eagle(MEM) (2225320, Gibco, Baltimore, MD, USA) and F-12 (2209586, Gibco, Baltimore, MD, USA) at equal percent, with the supplement of 10% fetal bovine serum (2001003, Biological Industries, Beit HaEmek, Israel), 1% sodium pyruvate (2185865, Gibco, MD, USA) and 1% penicillin/streptomycin (2185865, Gibco, Baltimore, MD, USA), under the condition of 5% CO_2_ at 37 °C. While HEK-293T cell was stored in liquid nitrogen and maintained in the Dulbecco’s modified Eagle’s medium (11965092, Gibco, Maryland, USA), with the supplement of 10% fetal bovine serum and 1% penicillin/streptomycin and storage condition as described before.

Primary neurons were acquired in the cerebral cortex from neonatal Sprague Dawley (SD) rats according to the method previously reported [103]. Neonatal SD rats were purchased from Hubei Center for Disease Control and Prevention. Primary neuron cells were preserved in the Neurobasal medium (21103049, Gibco, MD, USA), supplied with 1% B27 (17504044, Gibco, MD, USA), 1% sodium pyruvate (2185865, Gibco, MD, USA) and 1% penicillin/streptomycin (2185865, Gibco, MD, USA), under the condition of 5% CO_2_ at 37 °C. Moreover, these experiments on animals got support from the Animal Ethics Committee of Wuhan University Center for Animal Experiment/A3 Laboratory, Wuhan University.

Cell transfection was performed by Neofect^TM^ DNA Transfection reagent (D210101, Neofect Biotech Co., Ltd., Beijing, China) due to the manufacturer’s instructions.

### 4.4. Reverse Transcription and Quantitative Real-Time PCR

According to the manufacturer’s instructions, total cellular RNA (after transfected and cultured for 24 h) and blood RNA were isolated from TRIzol reagent (15596018, Invitrogen, California, USA) and TRIzol LS reagent (10296028, Invitrogen, California, USA) separately. Then 0.5 μg RNA was used to obtain cDNA through the ReverTra kit (FSQ-301; Toyobo, Osaka, Japan). The mRNA expression level was detected in the detector (T100, Bio-Rad, California, USA) by utilizing a 2× SYBR Green qPCR Mix (2992239AX, Aidlab Biotechnologies Co. Ltd., Beijing, China). Glyceraldehyde-3-phosphate dehydrogenase (GAPDH) was the internal reference, and the mRNA expression value was calculated through the method of 2^−ΔΔCt^. All primers were designed by oligo7 and listed in Supplementary Table S3.

### 4.5. Western Blotting Analysis

After transfected and cultured for 48 h, cells were washed with phosphate-buffered saline (PBS) and lysed by M-PER reagents (78501, Pierce Chemical, IL, USA) containing protein inhibitors (ab201119, Abcam, Cambridge, UK)). Protein quantification was achieved by Pierce TM BCA Protein Assay (UD281372; Thermo Fisher Scientific, Waltham, MA, USA). Samples with loading buffer were loaded onto a 10% SDS-PAGE, then electrotransferred to the PVDF membrane (IPVH00010; Amersham Biosciences, NJ, USA). Then membranes were cut due to molecular weight and incubated with primary antibodies at 4 °C overnight. The membranes were washed with TBST and hybridized with secondary antibodies for one hour at room temperature. Finally, ECL chemiluminescence solution (SW2030, Biosharp, Hefei, China) exposure made the protein band visualized through an automatic chemiluminescence system (5200, Tanon, Shanghai, China). Relative protein expression levels were qualified to GAPDH, and data were obtained from independent triplicate samples. Antibodies used in this study were listed in Supplementary Table S4.

### 4.6. Subcellular Fractionation

The separation of nuclear and cytoplasmic fractions was conducted with the method described [104]. In brief, SH-SY5Y cells were harvested and washed with PBS twice. After resuspending and homogenization, cells were centrifuged at 400× *g* for 15 min at 4 °C. The cytoplasmic fraction of the supernate was added with 1 mL Trizol agent for cytoplasmic RNA extraction. The nuclear RNA was separated after being washed with the nuclear isolation buffer. The cytoplasmic RNA and nuclear RNA were separated with the Trizol agent manufacturer’s instructions. The internal reference of the nuclear and cytoplasmic fraction was U6 and RPS14, respectively.

### 4.7. ELISA

According to the manufacturer’s instructions, the human IFN-β expression in serum and culture supernatant was tested by ELISA kit (MM-51652H1, Meiman Industrial Co. Ltd., Yancheng, China). The IFN-β concentration was calculated due to its absorbance at 450 nm wavelength by a spectrophotometer (FC357, Thermo Fisher Scientific, MA, USA).

### 4.8. Luciferase Assay

Luciferase activity was measured through Dual Glo Luciferase Assay System (E1960, Promega, Fitchburg, WI, USA) due to the manufacturer’s instructions. SH-SY5Y cells were cultured in the cell culture plate of 24 wells. Co-transfection of the plasmid and target gene in SH-SY5Y cells was performed to test luciferase activity after 24 h under the condition of 5% CO_2_ at 37 °C. The Renilla luciferase reporter plasmid (pRL-TK, Promega) was used as the internal control.

### 4.9. Co-Immunoprecipitation Assay

Co-immunoprecipitation was carried out as previously described [20]. The plasmids pENTER-N-FLAG-cGAS and pXJ40-HA-ENV, negative control plasmids (pENTER-N-FLAG and pXJ40-HA) were transfected into HEK-293T cells at the ratio of 1:1 (5 μg + 5 μg) in 100 mm cell culture dish and incubated for 48 h under the condition of 5% CO_2_ at 37 °C. After washing and lysing, cells were centrifuged at 12,000 rpm for 5 min to get the supernatant. Next, the supernatant was mixed with anti-Flag (L-1011, Bio-linkedin, Shanghai, China), anti-HA magnetic beads (L-1009, Bio-linkedin, Shanghai, China) and negative control mouse IgG antibody (dilution 1:200, AC011, ABclonal Technology, Wuhan, China) separately and maintained at 4 °C overnight. Then, the supernatant containing IgG was mixed with protein A/G magnetic beads (L-1004, Bio-linkedin, Shanghai, China) and warmly rotated for 2 h at room temperature. Finally, magnetic beads were washed with cell lysis buffer (P0013, Beyotime, Shanghai, China) and detected by western blotting.

### 4.10. Cell Proliferation Assay

Cell proliferation was performed with the cell counting kit 8 (CCK-8) (ZP328-1, Zomanbio, Beijing, China) according to the manufacturer’s instructions. Cells were transfected with plasmids at 96-well plates and incubated for 48 h. The absorbance value at 450 nm through a micro-plate reader after 10 μL CCK8 agent was added to the medium for 45 min.

### 4.11. Flow Cytometry

After plasmids transfection, SH-SY5Y cells were performed with Annexin V-FITC/PI Apoptosis Assay Kit (ZP327, Zomanbio, Beijing, China) according to the manufacturer’s instructions. The apoptosis rate was measured by Cytoflex S (Beckman Coulter, Brea, CA, USA) and analyzed via Cytexpert (Beckman Coulter, Brea, CA, USA).

### 4.12. Statistical Analyses

GraphPad Prism 5 was mainly used for data analysis through Student’s t-tests and one-way analysis of variance, with a significance value of *p* < 0.05. In addition, HERV-W ENV, linc01930, and IFN-β expression in schizophrenia patients and healthy controls were analyzed via median analyses and Mann-Whitney U analyses, with correlation analyses via Spearman’s rank correlation. Data were counted at least from three replicates and displayed as the mean ± SD. * *p* < 0.05; ** *p* < 0.01; *** *p* < 0.0001.

## 5. Conclusions

**Figure 9:**
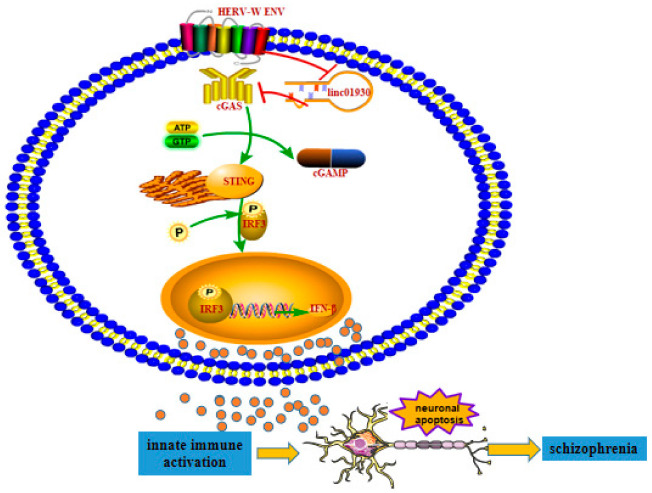
The potential role of HERV-W ENV to trigger neuronal apoptosis via innate immune activation in schizophrenia. The decreased linc01930 was negatively correlated with increased HERV-W ENV and IFN-β in schizophrenia. HERV-W ENV repressed linc01930 expression via its promoter activity. HERV-W ENV activated cGAS and STING expression and elevated IRF3 phosphorylation, while linc01930 functioned as a negative regulator to HERV-W ENV-induced cGAS and STING expression and IRF3 phosphorylation. In addition, linc01930 was involved in regulating the cGAS/STING signaling pathway induced by HERV-W ENV. Moreover, HERV-W ENV activated IFN-β expression via its promoter activity, while linc01930 inhibited linc01930 expression via its promoter activity. Furthermore, HERV-W ENV mediated the increased cGAS and IFN-β expression and neuronal apoptosis by regulating linc01930 expression. Thus, Innate immune activation might contribute to the etiology of schizophrenia.

In this paper, we found decreased linc01930 in the serum of schizophrenia, which was negatively correlated with HERV-W ENV, suggesting the promising role of linc01930 as a biomarker. We also found the increased IFN-β in schizophrenia, with a negative correlation to linc01930 and a positive correlation to HERV-W ENV. In vitro experiments demonstrated that HERV-W ENV inhibited linc01930. Additional studies suggested that linc01930, with nuclear location and noncoding ability, counteracted antiviral innate immunity, restrained neuronal apoptosis and exerted cell proliferation in neuron cells. Further studies proclaimed that HERV-W ENV induced innate antiviral immunity and neuronal apoptosis through cGAS/STING/IFN-β signaling pathway (Figure 9).

## Figures and Tables

**Table 1:** The RNA level of linc01930 in the blood of Healthy controls and Schizophrenia patients.

Healthy Control (N = 26)		Schizophrenia (N = 21)	
mean	1.62	mean	0.42
median	0.40	median	0.05
Standard deviation	2.44	Standard deviation	0.93
skewness	2.33	skewness	3.43
Standard Error of skewness	0.46	Standard Error of skewness	0.50
Range	10.25	Range	4.10
Minimum	0.0181	Minimum	0.0003
Maximum	10.27	Maximum	4.10

Clinical data were analyzed by median analyses.

**Table 2:** The concentration of IFN-β in the blood of Healthy controls and Schizophrenia patients.

Healthy Controls (N = 26)		Schizophrenia (N = 21)	
mean	40.89	mean	70.72
median	31.02	median	52.13
Standard deviation	26.04	Standard deviation	43.49
skewness	1.96	skewness	1.591
Standard Error of skewness	0.46	Standard Error of skewness	0.50
Range	104.59	Range	134.08
Minimum	18.01	Minimum	33.78
Maximum	122.60	Maximum	167.86

Clinical data were analyzed by median analyses.

**Table 3:** The mRNA level of HERV-W ENV in the blood of Healthy controls and Schizophrenia patients.

Healthy Controls (N = 26)		Schizophrenia (N = 21)	
mean	0.75	mean	5.13
median	0.23	median	1.65
Standard deviation	1.21	Standard deviation	9.90
skewness	3.01	skewness	2.92
Standard Error of skewness	0.456	Standard Error of skewness	0.501
Range	5.56	Range	35.44
Minimum	0.02	Minimum	0.85
Maximum	5.58	Maximum	36.30

Clinical data were analyzed by median analyses.

**Table 4:** The consistency of HERV-W ENV and linc01930 expression in Schizophrenia patients.

Schizophrenia Patients	HERV-W ENV (+)	HERV-W ENV (−)	Consistency Ratio
linc01930 (+)	11	6	57.1%
linc01930 (−)	3	1	

HERV-W ENV (+): the expression of HERV-W ENV above 1.2431; HERV-W ENV (−): the expression of HERV-W ENV below 1.2431; linc01930 (+): the expression of linc01930 below 0.6364; linc01930 (−): the expression of linc01930 above 0.6364. Clinical data were analyzed by median analyses.

**Table 5:** The consistency of HERV-W ENV and IFN-β expression in Schizophrenia patients.

Schizophrenia Patients	HERV-W ENV (+)	HERV-W ENV (−)	Consistency Ratio
IFN-β (+)	9	2	66.7%
IFN-β (−)	5	5	

HERV-W ENV (+): the expression of HERV-W ENV above 1.2431; HERV-W ENV (−): the expression of HERV-W ENV below 1.2431; IFN-β (+): the expression of IFN-β above 51.4044 ng/L; IFN-β (−): the expression of IFN-β below 51.4044 ng/L. Clinical data were analyzed by median analyses.

**Table 6:** The consistency of linc01930 and IFN-β expression in Schizophrenia patients.

Schizophrenia Patients	linc01930 (+)	linc01930 (−)	Consistency Ratio
IFN-β (+)	8	3	42.8%
IFN-β (−)	9	1	

linc01930 (+): the expression of linc01930 below 0.6364; linc01930 (−): the expression of linc01930 above 0.6364; IFN-β (+): the expression of IFN-β above 51.4044 ng/L; IFN-β (−): the expression of IFN-β below 51.4044 ng/L. Clinical data were analyzed by median analyses.

## Data Availability

All data is available from the corresponding author upon request.

## Supplementary Materials

The following supporting information can be downloaded at: https://www.mdpi.com/article/10.3390/ijms24033000/s1.
