# Supplementary material for: HERV-W ENV Induces Innate Immune Activation and Neuronal Apoptosis via linc01930/cGAS Axis in Recent-Onset Schizophrenia
Source: Int J Mol Sci. 2023 Feb 3;24(3):3000. doi: 10.3390/ijms24033000 (PMC9917391; doi:10.3390/ijms24033000)
Supplement: Supplementary file 1 [file ijms-24-03000-s001.zip › ijms-2164478-supplementary.pdf]

Supplementary Table S1.

Comparison of demographic and clinical characteristics between Schizophrenia and Healthy controls in Chinese (yellow race)

**characteristics**	**Recent-onset schizophrenia (n=21)**	**Healthy controls (n=26)**	***P* value**
Age(year)	44.0±12.32	42.46±10.28	0.708
Education(year)	10.00±3.62	10.65±3.82	0.665
BMI(body mass index)	21.06±2.74	20.56±3.96	0.460
Gender(male/female)	8/13	10/16	0.980
Smokers	6(28.6%)	7(26.9%)	0.9

All values are means ± SDs unless otherwise indicated. Age, Education and BMI were calculated by the Mann-Whitney U test. Gender and Smokers were calculated by the chi-square test.

Supplementary Table S2

PCR primer for plasmid construction

**plasmid**	**Oilgo sequence(5′>3′)**
pcDNA3.1-linc01930	F-CCGGAATTCCCAAAAGTGGAAACAATTCA
	R-CGCGGATCCTTGCTGTTTAAATCATATTT
pGL3-Basic-linc01930-promoter	F-CGGGGTACCGAAAAAGTTCCACTTCACCAC
	R-CCCAAGCTTCTATTAAGAATAATGCTGCT
pXJ40-HA-HERV-W-ENV	F-CCGGAATTCGCCACCATGGCCCTCCCTTATC
	R-CCCAAGCTTCTAACTGCTTCCTGCTGAAT
pENTER-N-FLAG-cGAS	F-CGGGGTACCATGCAGCCTTGGCACGGAAAGGC
	R-CCCAAGCTTAAATTCATCAAAAACTGGAAACT

Supplementary Table S3.

Primer sequence for qRT-PCR

**Target gene**	**Oilgo sequence(5′>3′)**	**Length (bp)**

Human-U6	F-CTCGCTTCGGCAGCACA	96
	R-AACGCTTCACGAATTTGCGT	
Human-RPS14	F-GTGTGACTGGTGGGATGAAGGTAA	127
	R-AGTTTGATGTGTAGGGCGGTGAT	
Human-linc01930	F-GTATGACTTTATAGCCGTGA	130
	R-CTTAAACTGACGACCCTGA	
Human-ENV	F-CCATGCCGCTGTATGACCAG	109
	R-GGGTTCCCTTAGAAAGACTCCT	
Human-cGAS	F-GGGAGCCCTGCTGTAACACTTCTTAT	187
	R-CCTTTGCATGCTTGGGTACAAGGT	
Human-STING	F-ACTGTGGGGTGCCTGATAAC	198
	R-TGGCAAACAAAGTCTGCAAG	
Human-IFN-β	F-GCTTGGATTCCTACAAAGAAGCA	166
	R-ATAGATGGTCAATGCGGCGTC	
Human-GAPDH	F-ATGACATCAAGAAGGTGGTG	177
	R-CATACCAGGAAATGAGCTTG	
Mouse-cGAS	F-CAGGAAGGAACCGGACAAGC	134
	R-CCGACTCCCGTTTCTGCATT	
Mouse-STING	F-GCCCCAGCAAAACATCGAC	187
	R-CTGCTCAAGCCGATCCTCC	
Mouse-IFN-β	F-GCCCTCTCCATCAACTATAAGCA	123
	R-GATCTTGAAGTCCGCCCTGT	
Mouse-GAPDH	F-GCATCTTCTTGTGCAGTGCC	105
	R-GAGAAGGCAGCCCTGGTAAC	

Supplementary Table S4.

Antibodies and dilution

Antibody	brand	catalog	dilution

ERVW-1	ABclonal	A16522	1:1000 (WB)
cGAS	ABclonal	A8335	1:1000 (WB)
STING	ABclonal	A3262	1:1000 (WB)
IFNB1	ABclonal	A1575	1:1000 (WB)
P-IRF3	Abcam	AB76493	1:1000 (WB)
GAPDH	ABclonal	AC002	1:10000 (WB)
GFP-Tag	ABclonal	AE012	1:10000 (WB)
IgG-HRP	ABclonal	AS003	1:5000 (WB)
IgG-HRP	ABclonal	AS014	1:5000 (WB)
IgG-HRP	ABclonal	AS061	1:5000 (WB)

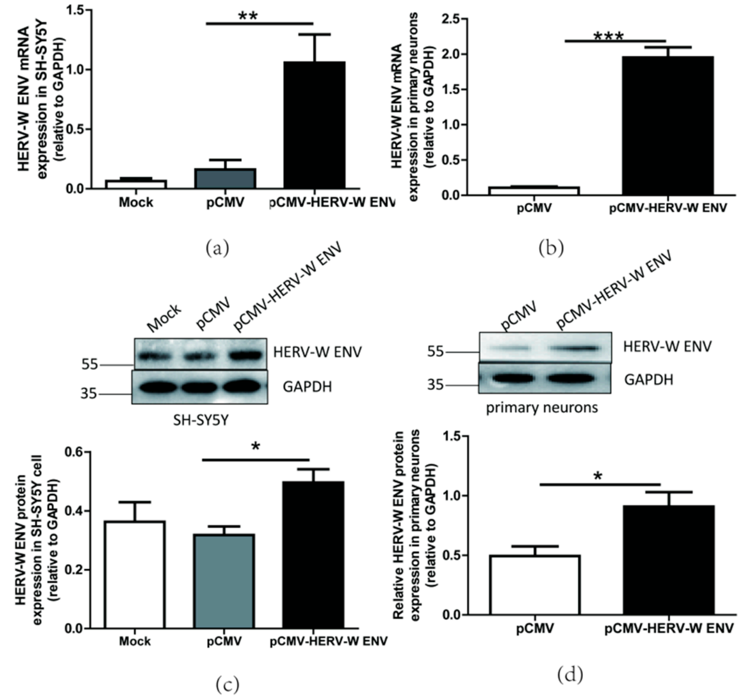

Supplementary Figure S1. Transfection efficiency of HERV-W ENV in neuronal cells. (a, b) The HERV-W ENV mRNA levels in HERV-W ENV transfected SH-SY5Y cells and primary neurons using qRT-PCR. (c, d) The HERV-W ENV protein levels in HERV-W ENV transfected SH-SY5Y cells and primary neurons using western blotting. Statistical analysis was performed by one-way analysis of variance (ANOVA). *$P < 0.05$; **$P < 0.01$.

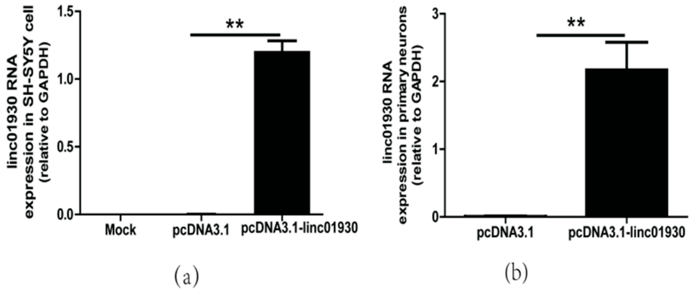

Supplementary Figure S2. Transfection efficiency of linc01930 in neuronal cells. (a) The linc01930 RNA levels in linc01930 transfected SH-SY5Y cells using qRT-PCR. (b) The linc01930 RNA levels in linc01930 transfected primary neurons using qRT-PCR. Statistical analysis was performed by one-way analysis of variance (ANOVA). *$P < 0.05$; **$P < 0.01$.

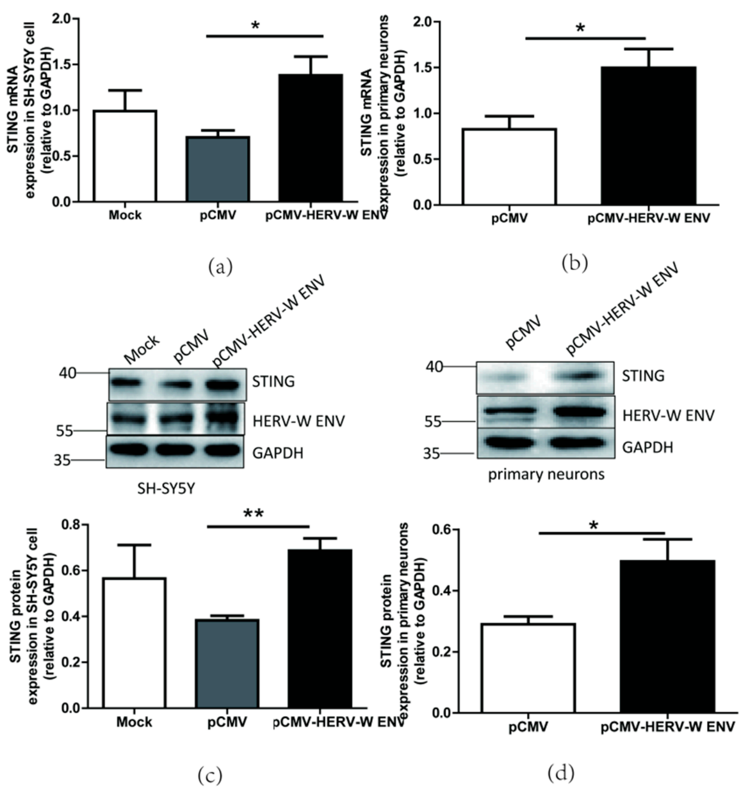

Supplementary Figure S3. HERV-W ENV activated STING expression in neuronal cells. (a, b) Respectively STING mRNA levels in HERV-W ENV transfected SH-SY5Y cells and primary neurons using qRT-PCR. (c, d) Respectively represent STING protein expression in HERV-W ENV transfected SH-SY5Y cell and primary neurons using western blotting. Statistical analysis was performed by one-way analysis of variance (ANOVA). *$P < 0.05$; **$P < 0.01$.

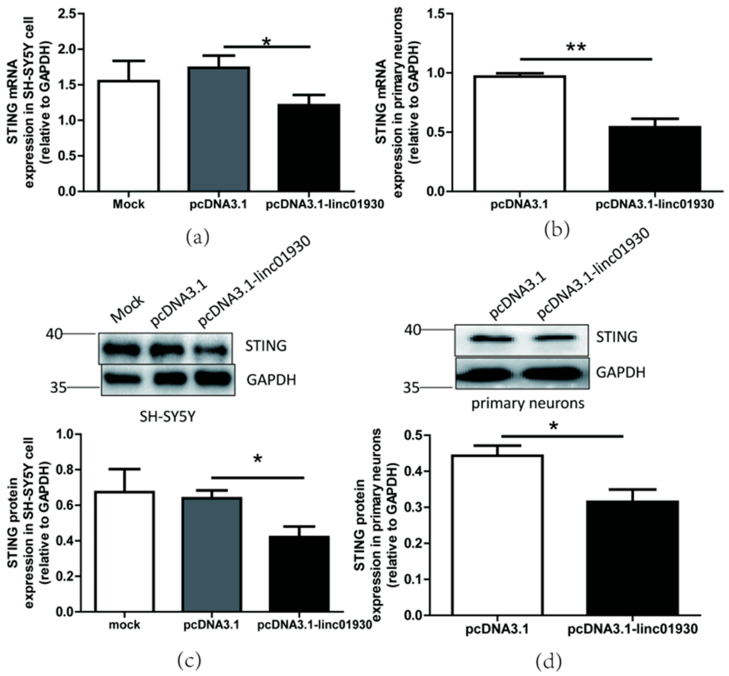

Supplementary Figure S4. Linc01930 inhibited STING expression in neuronal cells. (a, b) Respectively STING mRNA levels in linc01930 transfected SH-SY5Y cells and primary neurons using qRT-PCR. (c, d) Respectively represent STING protein expression in linc01930 transfected SH-SY5Y cell and primary neurons using western blotting. Statistical analysis was performed by one-way analysis of variance (ANOVA). **P* < 0.05; ***P* < 0.01.

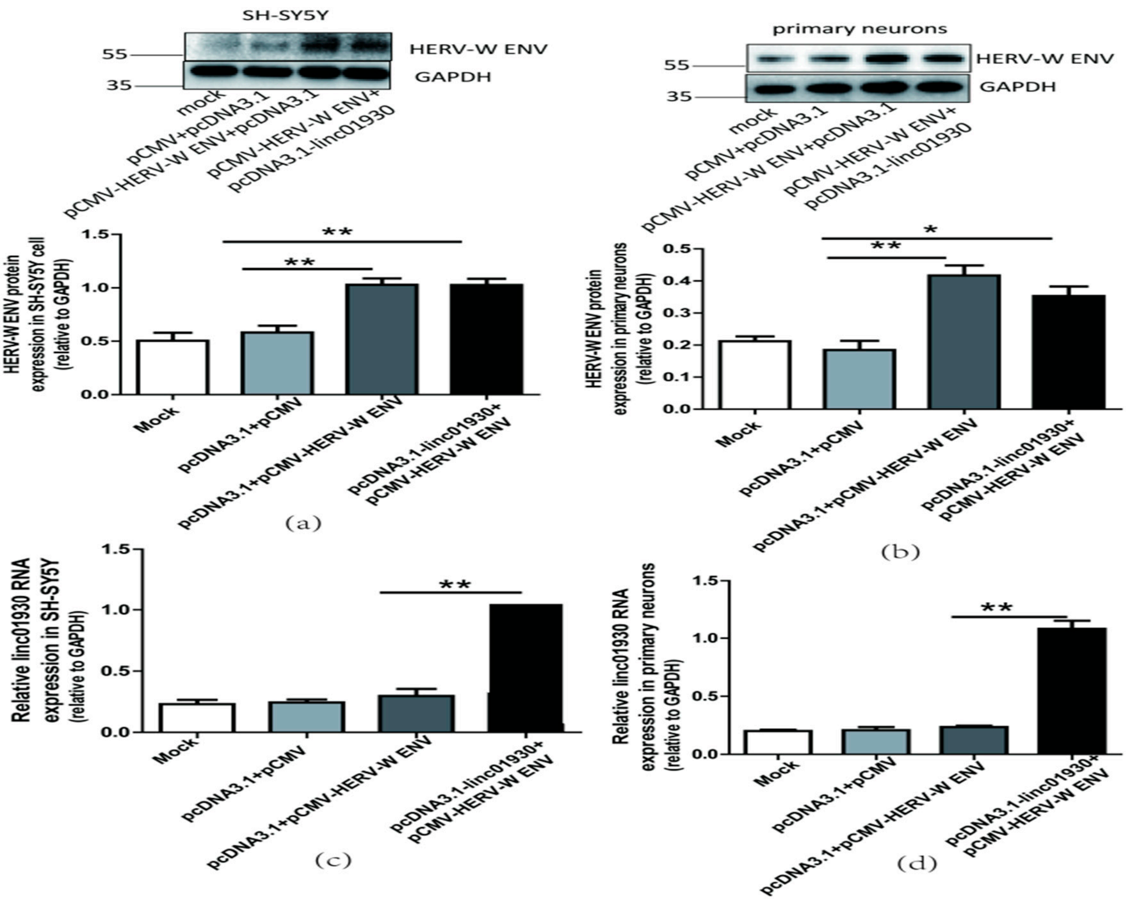

Supplementary Figure S5. Expression levels of HERV-W ENV and linc01930 in co-transfected neuronal cells. (a, b) Respectively representative western blots for HERV-W ENV in SH-SY5Y cell and primary neurons after co-transfected with linc01930 and HERV-W ENV. (c, d) Respectively representative linc01930 RNA levels in SH-SY5Y cell and primary neurons after co-transfected with linc01930 and HERV-W ENV. Statistical analysis was performed by one-way analysis of variance (ANOVA). *$P < 0.05$; **$P < 0.01$.

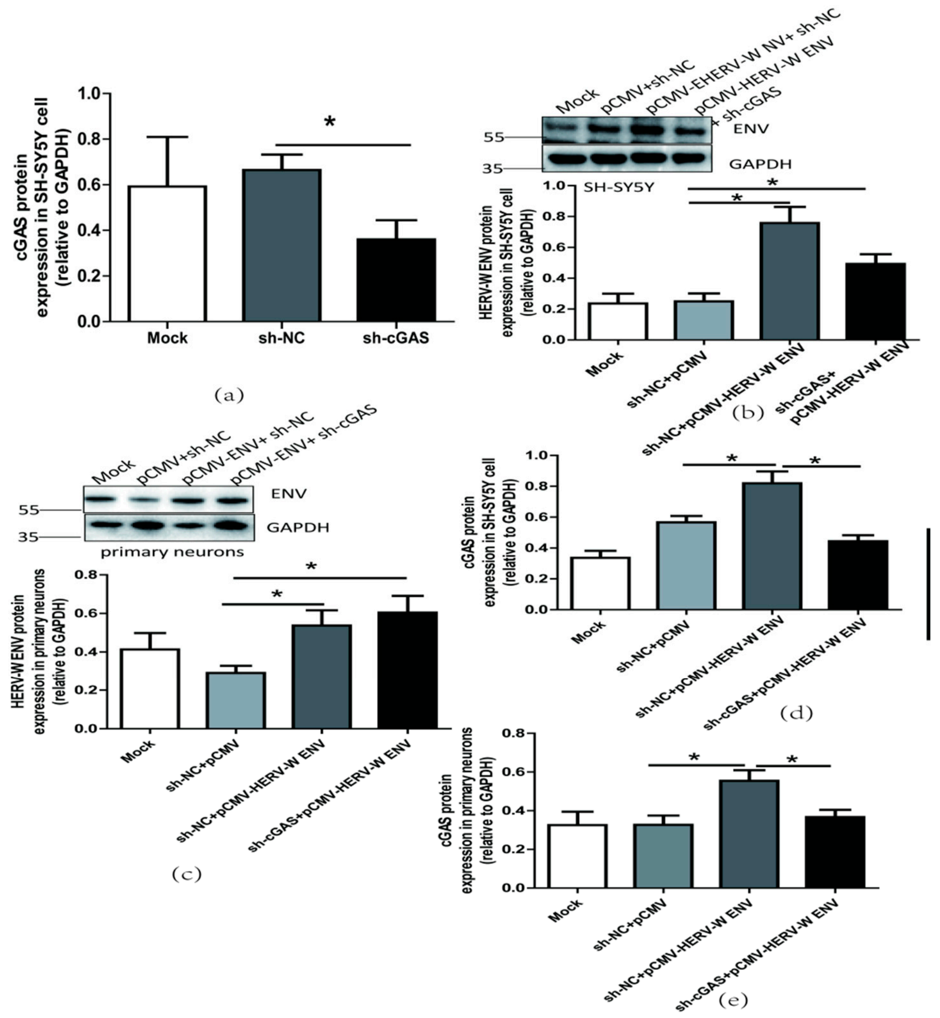

Supplementary Figure S6. Expression levels of HERV-W ENV and cGAS in co-transfected neuronal cells. (a) Representative western blots for cGAS in SH-SY5Y cells after cGAS knockdown. (b, c) Respectively representative western blots for HERV-W ENV in SH-SY5Y cells and primary neurons after co-transfected with sh-cGAS and HERV-W ENV. (d, e) Respectively representative western blots for cGAS in SH-SY5Y cell and primary neurons after co-transfected with sh-cGAS and HERV-W ENV. Statistical analysis was performed by one-way analysis of variance (ANOVA). *$P < 0.05$.
